# LncRNA EBLN3P promotes the progression of osteosarcoma through modifying the miR-224-5p/Rab10 signaling axis

**DOI:** 10.1038/s41598-021-81641-6

**Published:** 2021-01-21

**Authors:** Shuhong Dai, Ning Li, Ming Zhou, Yue Yuan, Ding Yue, Tao Li, Xiaowei Zhang

**Affiliations:** 1 Department of Cardiac Intensive Care Unit, Zibo Central Hospital, Zibo, Shandong Province China; 2 Department of Combination of Chinese Traditional and Western Medicine, Zibo Central Hospital, Zibo, Shandong Province China; 3 Department of Orthopedic Surgery, Zibo Central Hospital, Zibo, Shandong Province China; 4 Experimental Center of Medical Biology, School of Basic Medical Sciences, Jilin University, Changchun, China; 5 Department of Pathogen Biology, The Key Laboratory of Zoonosis, Chinese Ministry of Education, College of Basic Medicine, Jilin University, Changchun, 130021 Jilin China; 6 Center for Translational Medicine, Zibo Central Hospital, Zibo, Shandong Province China

**Keywords:** Cancer prevention, Cancer screening, Metastasis, Oncogenes

## Abstract

The treatment of patients with advanced-stage osteosarcoma represents a major challenge, with very few treatments currently approved. Although accumulating evidence has demonstrated the importance of lncRNAs in osteosarcoma, the current knowledge on the functional roles and molecular mechanisms of lncRNA endogenous born avirus-like nucleoprotein (EBLN3P) is limited. At present, the expressions of EBLN3P and miR-224-5p in osteosarcoma tissues were quantified by reverse transcription-quantitative PCR assay, and the expression of Ras-related protein 10 (Rab10) in osteosarcoma tissues was quantified by immunohistochemistry and western-blotting. The bioinformatics prediction software ENCORI was used to predict the putative binding sites of EBLN3P, Rab10 and miR-224-5p. The regulatory role of EBLN3P or miR-224-5p on cell proliferation, migration and invasion ability were verified by Cell Counting Kit-8, wound healing and Transwell assays, respectively. The interaction among EBLN3P, miR-224-5p and Rab10 were testified by luciferase. The increased expression of EBLN3P and Rab10 and decreased expression of miR-224-5p were observed in osteosarcoma tissues and cell lines. Besides, the overexpression of EBLN3P or knockdown of miR-224-5p were revealed to promote the proliferation, migration and invasion of osteosarcoma cells. Bioinformatics analysis and luciferase assay revealed that EBLN3P could directly interacted with miR-224-5p to attenuate miR-224-5p binding to the Rab10 3′-untranslated region. Furthermore, the mechanistic investigations revealed activation of the miR-224-5p/Rab10 regulatory loop by knockdown of miR‐372-3p or overexpression of Rab10, thereby confirming the in vitro role of EBLN3P in promoting osteosarcoma cell proliferation, migration and invasion. To the best of our knowledge, the present study is the first to demonstrate that EBLN3P may act as a competitive endogenous RNA to modulate Rab10 expression by competitive sponging to miR-224-5p, leading to the regulation of osteosarcoma progression, which indicates a possible new approach to osteosarcoma diagnosis and treatment.

## Introduction

Osteosarcoma is the most common primary bone tumor in children and adolescents^1^. Although the prognosis of osteosarcoma has improved significantly with the improvement in treatment methods, the efficacy of treatment remains unsatisfactory^2^. The main causes for the poor prognosis of osteosarcoma are recurrence and metastasis, and the 5-year survival rate after recurrence and metastasis is < 20%^3^. Early diagnosis and timely treatment of osteosarcoma are crucial for improving the prognosis of this tumor^4^. Recently, the American Cancer Society's ‘Blueprint 2030 for Cancer Prevention and Mortality Reduction’ have highlighted three key components of cancer mortality reduction: prevention, screening and treatment^5^. Tumor markers are important tools in the prevention and screening of malignant tumors, but only few mature biomarkers are currently used to guide clinical practice^6^. Therefore, there is an urgent need for new stable and reliable biomarkers in tumor research.

Long non-coding RNAs (lncRNAs) are non-protein-coding RNA molecules with a length of > 200 nt^7^. To date, a large number of lncRNAs have been found to be specifically expressed in various tumor tissues, and the mechanism of lncRNAs in gene regulation and tumorigenesis is gradually being uncovered^8^. MicroRNAs (miRNAs) are encoded by endogenous genes with a length of about 22 nucleotides, which possess multiple vital regulating roles in a variety of cells^9,10^. LncRNA is considered to be a competitive endogenous RNA (ceRNA) that can affect gene silencing caused by miRNA by binding to the miRNA through response elements^11^. For instance, lncRNA-PCAT1 has been found to regulate cell proliferation by combining with miRNA in esophageal squamous cell carcinoma, and may be used as a new serum-based biomarker for esophageal squamous cell carcinoma^12^. MicroRNA-224 (miR-224), as a member of the micro-RNAs family, is involved in the process of tumor cell proliferation, differentiation, apoptosis, invasion and metastasis, and plays an important role in the occurrence and development of human tumors^13^. miR-224 is highly expressed in various human tumors, such as liver cancer, breast cancer, colorectal cancer, cervical cancer, etc.^14,15^. In recent years, abnormal expression of miR-224 has been found to be related to the clinical characteristics and prognosis of tumors such as prostate and ovarian cancers^16,17^. However, little is known about between miR-224 and osteosarcoma.

Pseudogene is a term used to describe a type of non-coding gene that is very similar to the coding gene, but cannot produce a functional protein. Some pseudogenes can transcribe ncRNAs^18^, these ncRNAs, with a length of > 200 nt, are a class of lncRNAs^19^. Recently, with the improved understanding of the regulatory effect of lncRNAs on gene expression, the roles of pseudogene-derived lncRNAs in different tumors have been attracting increasing attention^20^. It was demonstrated that lncRNA-PTENP1, which is derived from a pseudogene, combines with miR-17 to regulate the expression of PTEN, a tumor suppressor gene, in order to inhibit the progression of bladder cancer^21^. In addition to the regulation of homologous genes, it was observed that pseudogenes can also affect the occurrence and development of tumors by regulating non-homologous genes^22^. For example, rp11-564d11.3 may act as a ceRNA to target vascular endothelial growth factor A as an oncogene^23^.

Ras-related proteins (Rabs) are a family of GTP-binding proteins (GTPases), and are among the most abundant proteins in exosomes^24^. Rabs are molecules necessary for vesicular movement and plasma membrane docking, and play an important role in the formation, transport, adhesion, anchoring and fusion of vesicles^25^. It is considered to be a molecular switch for vesicular transport between organelles, and participates in mediating exosome secretion^26^. Recently, Liang et al. reported that lncRNA HOTAIR can promote tumor aggressive behavior by affecting the expression and localization of the Rab35 protein^27^. A recent study investigated the expression of Rab10 in osteosarcoma cells and its involvement in the regulation of cell proliferation and migration^28^. Hence, in our previous research, the ceRNA network regulating Rab10 was investigated using bioinformatics and verified by molecular biology technology, and it was predicted that the expression of Rab10 is regulated by the lncRNA EBLN3P derived from pseudogenes via binding with the miR-224. Herein, whether miR-224 takes part in mediating the functions of lncRNA EBLN3P in osteosarcoma cells has aroused our strong research interest. The aim was to determine whether EBLN3P can affect the malignant phenotype of osteosarcoma cells by regulating Rab10 protein expression.

## Materials and methods

### Clinical tissue sample collection

A total of 29 cases of osteosarcoma tumor specimens and 27 cases of non-neoplastic bone specimens were collected from patients who underwent surgery at Zibo Central Hospital Affiliated to Shandong University. None of the patients had received any anticancer therapy prior to tumor resection or had been diagnosed with any additional malignancies. Pathological staging was based on the UICC/AJCC TNM classification (8th edition, 2016). Written informed consent was obtained from all participants. If the child was under 18 years of age and the informed consent was provided by a parent or legal guardian. The collection of clinical specimens was approved by the Ethics Committee of Zibo Central Hospital Affiliated to Shandong University, according to the principles outlined in the Declaration of Helsinki.

### Reverse transcription-quantitative PCR (RT-qPCR) analysis

Total RNA was extracted and reverse-transcribed to cDNA using the PrimeScript RT Master Mix kit (Takara Bio, Inc.) according to manufacturer’s protocol. The gene expression was explored using FastStart Universal SYBR Master mix (Roche Diagnostics GmbH). The relative expressions were calculated by the 2−ΔΔCt method as previously prescribed^29^. The primer sequences are listed in Table 1.

**Table 1:** Primer sequences for quantitative real-time PCR.

	Forward sequence	Reverse sequence
EBLN3P	5′-CAGACTAAAGGATCAAGCGAGA-3ʹ	5′-ATCAATTGCCACAGGTTGAAGA-3ʹ
miR-224-5p	5′- GCCCCGACAGTCTAGATATGA -3ʹ	5′- GGATGCTGCTGCTAGAGGT -3ʹ
Rab10	5′- GGATACCTACGGAGCACGAG-3ʹ	5′- AGCCATCACACTTCTCCAGG-3ʹ
U6	5′-CTCGCTTCGGCAGCACA-3ʹ	5′-AACGCTTCACGAATTTGCGT-3ʹ
GAPDH	5′-CCAGGTGGTCTCCTCTGA-3ʹ	5′-GCTGTAGCCAAATCGTTGT-3ʹ

### Western blotting

Proteins were extracted from cells using RIPA protein extraction buffer (Beyotime Institute of Biotechnology), and were quantified by the Bradford assay as previously described^30^. The same amount up to 30ug of protein was subjected to 10% SDS-PAGE and transferred to PVDF membranes. After being blocked in 10% skimmed milk, the membranes were incubated with Rab10 polyclonal antibody (1:1000 dilution; cat. no. ab104859, Abcam) at 4 °C overnight, washed 3 times for 5 min and then incubated with the corresponding horseradish peroxidase (HRP)-conjugated anti-rabbit IgG (1:5000 dilution; cat. no. sc-2004 Santa Cruz Biotechnology, Inc.) at room temperature for 60 min. The protein bands were visualized with ECL Super Signal reagent (Pierce; Thermo Fisher Scientific, Inc.). The relative intensity of the bands was determined using Image J software (version 1.41o, Java 1.6.0_10, https://imagej.nih.gov/ij/, Wayen Rasband, National Institutes of Health). Where gels/blots are used in figures were compliance with the digital image and integrity policies (www.nature.com/srep/policies/index.html#digital-image).

### Immunohistochemistry (IHC) analysis

Routine hematoxylin and eosin staining were performed prior to IHC analysis as previously described^29^. Briefly, paraffin-embedded samples were cut into 3-μm sections, and then dewaxed with xylene and rehydrated for peroxidase (DAB) IHC staining. For antigen retrieval, the sections were heated at 97 °C for 20 min. Following a brief proteolytic digestion and peroxidase blocking, the sections were incubated with Rab10 polyclonal antibody (1:500 dilution; cat. no. ab104859, Abcam) overnight at 4 °C, and HRP/Fab polymer conjugate (cat. no. PV-6000-D, Zhongshan Golden bridge Biotechnology Co. Ltd.) was applied as secondary antibody (1:5,000 dilution) at room temperature for 60 min. Finally, the sections were stained with diaminobenzidine substrate and counterstained with hematoxylin. Two independent investigators semi-quantitatively evaluated Rab10 positivity without prior knowledge of the clinicopathological data. The final immunoreactivity scores (IRS) were assessed according to score of the percentage of positive cells (0, 0–5%; 1, 6–25%; 2, 26–50%; 3, 51–75%; and 4, 76–100% positive cells) plus the staining intensity score (0, no staining; 1, weak; 2, moderate; and 3, strong staining). Final IRS > 4 was accepted as strong positivity, while all others were considered to indicate weak positivity.

### Cell culture

The human fetal osteoblast cell line hFOB.1.19 and the osteosarcoma cell lines Saos2, MG63, 143B and U2OS were obtained from American Type Culture Collection and cultured in RPMI-1640 medium supplemented with 10% fetal bovine serum (FBS; Gibco BRL, Grand Island, NY) in a humidified incubator containing 5% CO_2_. The cells were passaged once every 2–3 days, and cells in the logarithmic growth phase were used for the subsequent experiments.

### Cell transfection and plasmid construction

EBLN3P overexpression plasmid (pcDNA-EBLN3P), the Rab10 overexpression plasmid (pcDNA-Rab10), mimics NC, miR-224-5p mimics, inhibitor NC, and miR-224-5p inhibitor were synthesized by Shanghai GenePharma Co., Ltd. The packaging construct including the vesicular stomatitis virus G (VSVG)‐expressing construct, pGCSIL-EGFP construct and pGCSIL-scramble construct were purchased from Genechem Biotech Co., Ltd. The cell transfections were performed using Lipofectamine 3000 (cat. no. 11668027, Invitrogen, Thermo Fisher Scientific, Inc.) according to the manufacturer’s instructions. Cells were gathered for further utilization after 48 h of cell transfection.

### Short hairpin RNA (shRNA) method

The shRNA-mediated knockdown was performed as previously described^29^. The 293 T cells were cultured to a 70–80% confluence in 6-well dishes before transfected with the aforementioned constructs using Lipofectamine 3000 (cat. no. 11668027, Thermo Fisher Scientific, Inc.). Then, the viral stocks were concentrated via ultracentrifugation at 38,000 rpm for 1 min and dissolved in Hanks’ balanced salt solution. The viral stocks were used to infect the osteosarcoma cells and fetal osteoblast cells at a multiplicity of infection of 200.

### Cell counting kit-8 (CCK-8) assay

Cell viability was measured using the CCK-8 assay (Dojingdo Molecular Technologies, Inc.) as previously described^31^. Briefly, cells (2 × 10^3^ cells per well) were cultured in 96-well plates and incubated at 37 °C for 24 h. Subsequently, 10 μl/well CCK-8 reagent was added, and the cells were incubated for a further 4 h. Finally, the absorbance of each group at 450 nm was measured using a microplate reader (Molecular Devices, LLC).

### Colony formation assay

After transfection for 24 h, the cells (5 × 10^4^ cells per well) were seeded into 6-well plates and incubated for 7 days. During this period, the medium was refreshed every 3 days. Subsequently, the cells were fixed with methanol at room temperature for 15 min and stained with giemsa reagent at room temperature for 5 min. Finally, the colonies were imaged and counted via a camera. Three separate experiments were performed for each assay.

### Wound healing assay

For cell migration ability determination, after the cells had grown to 90% confluence, and then the cells were serum-starved for 24 h to inhibit the cell proliferation. A wound was generated using a 10 μl pipette tip, and the culture medium was changed to remove the detached cells. After 24 h, the cells were visualized by light microscopy. The procedure was carried out as described previously^32^.

### Transwell assay

To determine cell invasion ability, the cells (8 × 10^2^ cells) were resuspended in serum-free medium and seeded onto the upper chamber of a Matrigel-coated Transwell insert (EMD Millipore). Complete medium supplemented with 10% FBS was added to the lower chamber. After 24 h, the upper surface of the membrane was wiped with a cotton swab, and the cells attached to the lower surface were fixed with 4% formaldehyde for 10 min at room temperature and stained with 1% DAPI (4′, 6-diamidino-2-phenylin-dole) solution for 10 min. The invaded cells were observed and counted under a light microscope. The procedure was carried out as described previously^32^.

### Dual-luciferase reporter assays

The procedure was carried out as described previously^33^. Wild-type (WT) and mutant (MUT) reporter plasmids of EBLN3P (EBLN3P-WT-luc and EBLN3P-MUT-luc), containing WT or MUT miR-224-5p mimics or mimics NC-binding sites were synthesized by GenePharma. The synthesized reporter plasmids were co-transfected with miR-224-5p mimics or mimics NC, respectively, by Lipofectamine 2000 (Invitrogen; Thermo Fisher Scientific, Inc.) when the cells reached 70% confluence. The luciferase activity was analyzed via the dual-luciferase reporter assay system.

### Bioinformatics ENCORI software

The bioinformatics ENCORI software^34^ (http://starbase.sysu.edu.cn/) has been used to systematically identify miRNA-mRNA and miRNA-lncRNA interaction networks. In this work, the ENCOR was used to predict the relationship and the binding sites among EBLN3P, miR-224-5p and Rab10.

### Statistical analysis

All the experimental results are expressed as the mean ± standard deviation, and each experiment was performed in triplicate. Statistical analyses and graphical depictions were performed using GraphPad Prism 5.0 (https://www.graphpad.com, GraphPad Software, Inc.). Student’s t-test or one-way analyses of variance (ANOVAs) were employed to evaluate the significance of the differences, as appropriate. The association between the survival rate of osteosarcoma patients and Rab10, miR-224-5p or EBLN3P expression levels was investigated using Kaplan–Meier survival curves and the log-rank test. Statistical significance was set at *P < 0.05 or **P < 0.01.

## Results

### EBLN3P and Rab10 are upregulated, while miR-224-5p is downregulated in osteosarcoma tissues and cell lines

**Figure 1:**
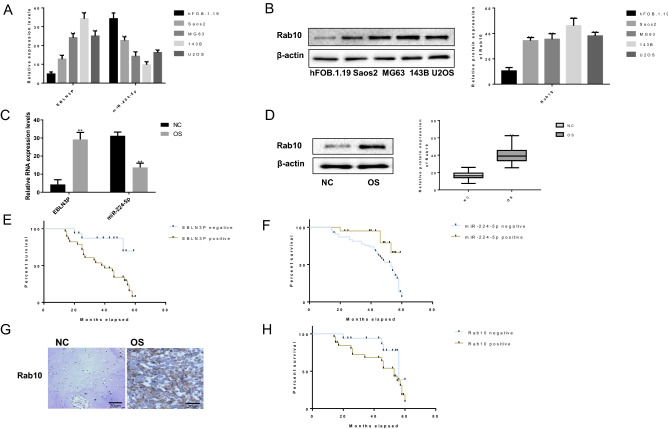
EBLN3P and Rab10 were upregulated while miR-224-5p was downregulated in osteosarcoma tissues and cell lines. (**A**) The expression levels of EBLN3P and miR-224-5p were assessed in osteosarcoma cell lines (Saos2, 143B, MG63, and U2OS) and the normal human fetal osteoblast cell line (hFOB.1.19) by RT-qPCR. (**B**) The western blotting results revealed that Rab10 was expressed at a higher level in the four osteosarcoma cell lines compared with the hFOB.1.19 cell line. (**C**) The expression patterns of EBLN3P and miR-224-5p were examined in osteosarcoma samples (OS) and non-cancerous tissues (NC) via RT-qPCR. (**D**) The expression patterns of Rab10 were examined in osteosarcoma samples (OS) and non-cancerous tissues (NC) via western blotting. (**E**) The associations between EBLN3P expression and the prognosis of patients with osteosarcoma were investigated. (**F**) Associations between miR-224-5p expression and the prognosis of patients with osteosarcoma. (**G**). The expression patterns of Rab10 were examined in osteosarcoma samples (OS) and non-cancerous tissues (NC) via IHC. (**H**) Associations between Rab10 expression and the prognosis of patients with osteosarcoma. *EBLN3P* endogenous bornavirus-like nucleoprotein, *OS* osteosarcoma samples, *NC* non-cancerous tissues, *Rab10* Ras-related protein 10, *RT-qPCR* reverse transcription-quantitative PCR, *IHC* immunohistochemistry.

To investigate the regulatory role of EBLN3P in osteosarcoma, it was first examined whether EBLN3P and miR-224-5p were dysregulated in osteosarcoma. To identify the potential genes targeted by miR-224-5p in osteosarcoma cells, the bioinformatics ENCORI software was used and Rab10 was identified as one of the target genes of miR-224-5p. The results of RT-qPCR analysis demonstrated that the RNA expression level of EBLN3P was expressed at higher levels in four osteosarcoma cell lines (Saos2, 143B, MG63 and U2OS) compared with those in the normal human fetal osteoblast cell line, while the RNA expression level of miR-224-5p exhibited the opposite trend (normalized to GAPDH, Fig. 1A). The results of western blot analysis demonstrated that the protein expression level of Rab10 were expressed at higher levels in four osteosarcoma cell lines compared with those in the normal human fetal osteoblast cell line (normalized to β-actin expression, Fig. 1B and Supplementary file). In addition, the expression levels of EBLN3P, miR-224-5p and Rab10 were accessed in osteosarcoma tissues and non-neoplastic bone tissues. As expected, the RNA expression of EBLN3P was higher and that of miR-224-5p was lower in osteosarcoma tissues compared with non-neoplastic bone tissues (normalized to GAPDH, Fig. 1C). Besides, the protein expression of Rab10 was higher in osteosarcoma tissues compared with non-neoplastic bone tissues (normalized to β-actin expression, Fig. 1D). Moreover, the associations between the expression of EBLN3P and miR-224-5p and the prognosis of osteosarcoma patients were investigated using Kaplan–Meier survival curves and the log-rank test. As shown in Fig. 1E,F,H, osteosarcoma patients with positive miR-224-5p expression exhibited longer overall survival compared with patients with negative miR-224-5p expression. In addition, osteosarcoma patients with positive EBLN3P or Rab10 expression had a shorter overall survival compared with that of patients with negative EBLN3P or Rab10 expression. Furthermore, the expression of Rab10 in osteosarcoma tissues was investigated via IHC analysis. As shown in Fig. 1G and Table 2, the expression of Rab10 was higher in osteosarcoma tissues (21/29) compared with that in non-neoplastic bone tissues (5/27), and the expression of Rab10 was found to be associated with pulmonary metastasis (P < 0.001) and TNM stage (AJCC) (P < 0.001). These data indicate that EBLN3P, miR-224-5p and Rab10 may play important roles in regulating the development of osteosarcoma.

**Table 2:** Expression of Rab10 and clinicopathological characteristics in OS patients.

Item	N	Rab10 ( +)	Rab10 (−)	*P*
Tumor tissue	29	21	8	< 0.001*
Non-neoplastic	27	5	22	
**Age (years)**				
≤ 19	16	9	7	0.764
> 19	13	8	5	
**Gender**				
Male	17	9	8	0.614
Female	12	7	5	
**Stage**				
IA–IIA	11	8	3	< 0.001*
IIB–III	18	7	11	
**Response to chemotherapy**				
Poor	10	5	5	0.126
Good	7	3	4	
NA (n = 11)	11			
**Pulmonary metastasis**				
+	22	14	7	< 0.001*
−	7	2	5	

*NA* not available.

*Statistical significance was found with the Chi-square test/Chi-Square Goodness-of-Fit Test.

### EBLN3P knockdown suppresses the proliferation, migration and invasion of osteosarcoma cells in vitro

**Figure 2:**
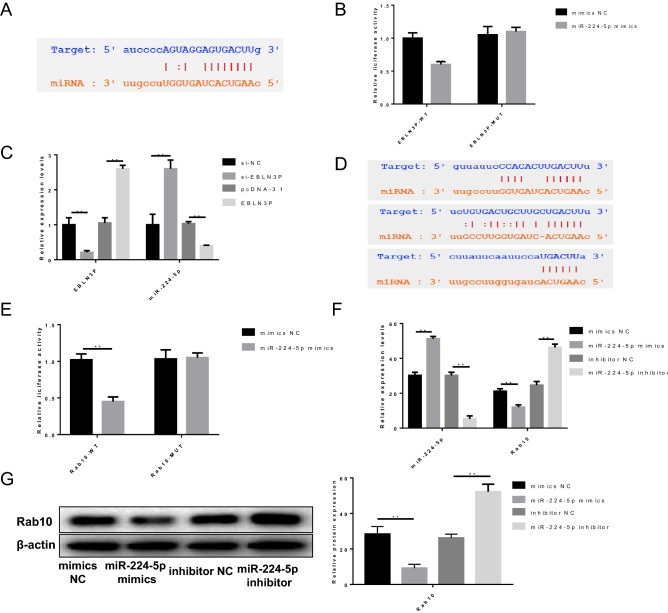
The interaction between EBLN3P, miR-224 and Rab10. (**A**) Bioinformatics analysis revealed that EBLN3P can directly interact with miR-224-5p. (**B**) Dual-luciferase reporter assays were performed to verify the effect of miR-224-5p on the luciferase signal of reporters containing EBLN3P. 143B cells were transfected with EBLN3P targeted shRNA (si-EBLN3P), negative control shRNA (si-NC), pcDNA3.1-EBLN3P overexpression vector (pcDNA-EBLN3P) and blank vector plasmid (pcDNA-3.1). (**C**) The expression level of EBLN3P and miR-224-5p was assessed via reverse transcription-quantitative PCR analysis. (**D**) Bioinformatics and luciferase assay revealed that miR-224-5p can directly bind to the Rab10 3′-UTR region. (**E**) The miR-224-5p mimics and luciferase reporter plasmids with wild-type or mutant Rab10 3′-UTR were co-transfected into 143B cells. Dual luciferase reporter gene assay was performed to verify the direct binding between miR-224-5p and Rab10. (**F**) 143B cells were transfected with mimics NC, miR-224-5p mimics, inhibitor NC, miR-224-5p inhibitor, and then reverse transcription-quantitative PCR was undertaken to evaluate the relative expression levels of miR-224-5p and Rab10. (**G**) Western blot analysis validated that overexpression of miR-224-5p significantly reduced Rab10 expression.

As mentioned above, the expressions of EBLN3P and miR-224-5p were negatively correlated in osteosarcoma cells and tissues (Fig. 1), suggesting the inhibitory effect of EBLN3P on miR-224-5p. Bioinformatics confirmed that EBLN3P directly interacted with miR-224-5p (Fig. 2A). Dual-luciferase reporter assays were performed to verify our hypothesis. Overexpression of miR-224-5p was achieved by transfecting 143B cells with miR-224-5p mimics. The luciferase assay results confirmed that transfection with miR-224-5p mimics significantly weakened the luciferase signal of reporters containing EBLN3P-WT, but had no effect on the activity of reporters containing EBLN3P-MUT (Fig. 2B). Next, 143B cells were transfected with EBLN3P shRNA (si-EBLN3P) and pcDNA3.1-EBLN3P overexpression vector (EBLN3P) to further investigate its possible impact on the aggressive behavior of osteosarcoma cells. The negative control shRNA (si-NC) and the blank vector plasmid (pcDNA-3.1) were used as knockdown and overexpression controls, respectively. Moreover, the expression level of EBLN3P and miR-224-5p were evaluated via RT-qPCR (Fig. 2C). The RT-qPCR results indicated a negative regulatory association between EBLN3P and miR-224-5p (Fig. 2C). Taken together, these results demonstrated that EBLN3P may serve as a ceRNA for miR-224-5p to inhibit miR-224-5p expression.

The effects of Rab10 on osteosarcoma cells and its interaction with miR-224-5p have yet to be elucidated. Bioinformatics analysis and luciferase assay confirmed that miR-224-5p can directly bind to the 3′-untranslated region (UTR) of Rab10 (Fig. 2D). Next, a dual-luciferase reporter gene assay demonstrated that the luciferase signal of cells co-transfected with miR-224-5p mimics and WT Rab10 vector was notably decreased compared with that of cells co-transfected with miR-224-5p mimics and MUT Rab10 vector (Fig. 2E). This indicated that miR-224-5p likely binds to the 3′-UTR of Rab10. Further RT-qPCR and western blot analysis validated that the overexpression of miR-224-5p significantly reduced the expression of Rab10, whereas knockdown of miR-224-5p significantly enhanced the expression of Rab10 at both the mRNA and protein levels in 143B osteosarcoma cells (Fig. 2F,G and Supplementary file). Taken together, these results indicate that miR-224-5p acts as the upstream regulator to adjust Rab10 expression.

### miR-224-5p inhibits the proliferation, migration and invasion of osteosarcoma cells via downregulating Rab10 in vitro

**Figure 3:**
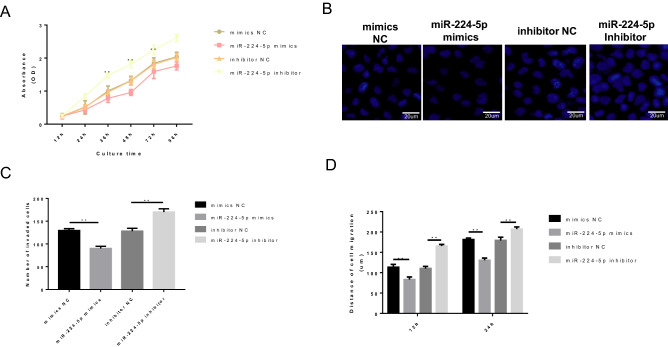
miR-224-5p inhibited the proliferation, migration and invasion of osteosarcoma cells via downregulating Rab10. (**A**) Cell proliferation was tested by the Cell Counting Kit-8 assay. (**B**) Cell invasion was detected by the Transwell assay. (**C**) Number of invading cells. (**D**) Cell migration was detected by the wound healing assay. *Rab10* Ras-related protein 10, *UTR* untranslated region.

The effects of miR-224-5p on the malignant behavior of osteosarcoma cells have yet to be elucidated. The CCK‐8 assay revealed an obviously decreased proliferation rate in the miR-224-5p mimics group and a significantly increased proliferation rate in the miR-224-5p inhibitor group, compared with the control group (Fig. 3A). The migration and invasion assays further verified this trend. The migration and invasion abilities of osteosarcoma cells were suppressed following miR-224-5p mimics transfection, whereas they were enhanced following inhibitor transfection (Fig. 3B–D). Collectively, these data indicate that miR-224-5p acts as a tumor suppressor in osteosarcoma cells, and it may suppress the malignant behaviors of 143B cells, including cell proliferation, migration and invasion.

### The knockdown of EBLN3P markedly suppressed the proliferation, invasion and migration of osteosarcoma cells

**Figure 4:**
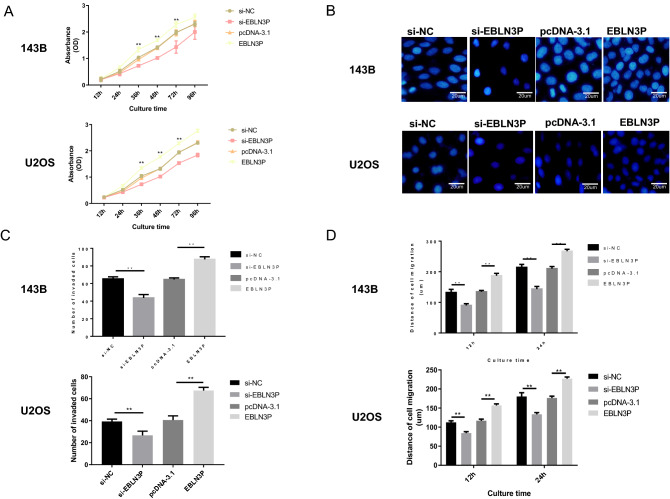
EBLN3P knockdown inhibited the proliferation, migration and invasion of osteosarcoma cells in vitro. (**A**) Cell proliferation was tested by Cell Counting Kit-8 assay. (**B**) Cell invasion were detected by Transwell assay. (**C**) The numbers of the invaded cells. (**D**) Cell migration was detected by the wound healing assay. *EBLN3P* endogenous bornavirus-like nucleoprotein.

The 143B and U2OS cells were transfected with EBLN3P targeted shRNA (si-EBLN3P), negative control shRNA (si-NC), pcDNA3.1-EBLN3P overexpression vector (pcDNA-EBLN3P) and blank vector plasmid (pcDNA-3.1). Moreover, the influence of the EBLN3P overexpression or knockdown on osteosarcoma 143B and U2OS cells were also explored. Our data revealed that the knockdown of EBLN3P markedly suppressed the proliferation, invasion and migration of osteosarcoma 143B and U2OS cells, which were enhanced by EBLN3P overexpression, as evidenced by CCK-8, Transwell and wound healing assays, respectively (Fig. 4A–D). Therefore, these results suggested that EBLN3P has an oncogenic potential and induces osteosarcoma cell proliferation, migration and invasion.

### *EBLN3P regulates osteosarcoma cells *via* the miR-224-5p/Rab10 pathway*

**Figure 5:**
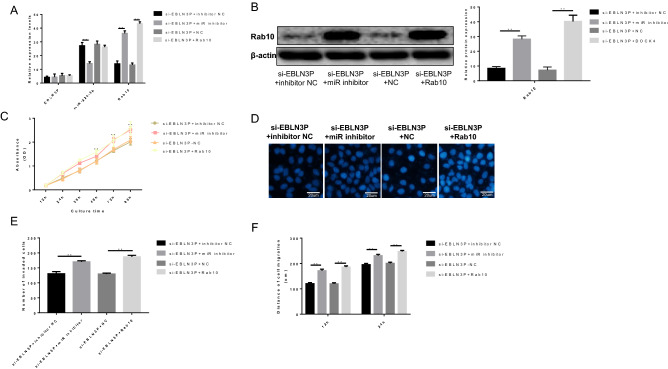
EBLN3P regulated the aggressive behavior of osteosarcoma cells via the miR-224-5p/Rab10 loop. (**A**,**B**) Overexpression of Rab10 was achieved via transfecting the cells with pcDNA3.1‐Rab10 or miR-224-5p inhibitor. The knockdown of EBLN3P markedly reduced cell proliferation, migration and invasion. However, co-transfection with si-EBLN3P and miR-224-5p inhibitor or Rab10 significantly increased cell proliferation, migration and invasion, in contrast to cells transfected with si-EBLN3P alone (Fig. 4C–F). *EBLN3P* endogenous bornavirus-like nucleoprotein, *Rab10* Ras-related protein 10.

Rescue experiments were performed to assess the effects of the EBLN3P-miR-224-5p-Rab10 pathway on 143B cell activity. Overexpression of Rab10 was achieved via transfecting the cells with pcDNA3.1‐Rab10 or miR-224-5p inhibitor (Fig. 5A,B and Supplementary file). The results demonstrated that knockdown of EBLN3P markedly reduced cell proliferation, migration and invasion. However, co-transfection with si-EBLN3P and miR-224-5p inhibitor or Rab10 significantly increased cell proliferation, migration and invasion compared with cells transfected with si-EBLN3P alone (Fig. 5C–F). Accordingly, these data suggested that the regulatory effects of EBLN3P were mediated through the miR-224-5p/Rab10 axis to promote the progression of osteosarcoma cells.

## Discussion

LncRNAs were initially identified in carcinogenesis due to their differential expression compared with normal tissues^35^. To date, a growing body of evidence indicates that the abilities of lncRNAs regulate complex cellular behaviors, such as cell growth and metastasis, are commonly deregulated in cancer, including osteosarcoma^36,37^. Although numerous of potential biomarkers have been reported, a specific diagnostic biomarker for osteosarcoma has not yet been confirmed^38^. EBLN3P is a novel lncRNA located on chromosome 9: 37,079,935–37,086,874 forward strand^39^. The present study demonstrated that EBLN3P was markedly upregulated in osteosarcoma tissues and cell lines. In vitro, functional assays indicated that the knockdown of EBLN3P suppressed osteosarcoma cell proliferation, migration and invasion, demonstrating the potential of EBLN3P as a therapeutic target for osteosarcoma; therefore, we next investigated the underlying mechanism in osteosarcoma cell lines.

The ceRNA theory was first proposed in 2011 and has since been widely accepted in the field of non-coding RNA research^40^. LncRNAs may serve as ceRNAs by sponging to miRNAs and inhibiting the downstream target gene^41^. Bioinformatics analyses revealed that there was a conserved binding site of miR-224-5p on EBLN3P. Therefore, it was inferred that EBLN3P could affect miR-224-5p via a ceRNA mechanism. It was validated that miR-224-5p exerted a reciprocal suppressive effect with EBLN3P expression, and knockdown of miR-224-5p induced the proliferation, migration and invasion of osteosarcoma cells in vitro. Importantly, the dual-luciferase assay further confirmed that EBLN3P directly interacted with miR-224-5p to reduce its expression, suggesting that EBLN3P serves as the miRNA sponge that binds to and regulates miR-224-5p expression.

The miRNAs control gene expression by binding to the 3′-UTR of the target gene, which causes mRNA cleavage or translational repression^42^. According to the results of previous and ongoing research, it was hypothesized that EBLN3P can affect the malignant phenotype of osteosarcoma cells by upregulating the expression of the Rab10 protein. To the best of our knowledge, this study is the first to explore the effect of EBLN3P on the malignant phenotype of osteosarcoma cells and its molecular mechanism of action, so as to provide a theoretical basis and data supporting EBLN3P as a molecular target for early diagnosis and metastasis control of osteosarcoma.

The present study provided evidence that Rab10 was overexpressed in osteosarcoma, and that its positive expression is associated with distant metastasis and a higher TNM stage. Osteosarcoma patients with positive Rab10 expression exhibited a shorter overall survival compared with patients with negative Rab10 expression. The present study demonstrated that Rab10 could promote the proliferation of osteosarcoma cells in vitro. It was also observed that the expression of Rab10 in distant metastases was higher compared with that in primary osteosarcoma tissues, and that Rab10 could increase the migration and invasion abilities of osteosarcoma cells in vitro and in vivo. In the present study, it was also observed that the expression of Rab10 was regulated via the EBLN3P/miR-224-5p axis through bioinformatics analyses; however, whether this compensatory mechanism exists in osteosarcoma requires further investigation. The dual-luciferase assays confirmed the direct interaction between miR-224-5p and Rab10. Further rescue experiments demonstrated that EBLN3P promoted the proliferation and metastasis of osteosarcoma cells via regulation of the miR-224-5p/Rab10 signaling axis.

Although we initially uncovered a novel downstream regulatory mechanism in osteosarcoma cells mediated by EBLN3P in vitro, further research is required to fully elucidate this complex mechanism. Future studies using mouse models will be needed to further verify this novel molecular mechanism and prove its therapeutic potential.

## Conclusion

In conclusion, to the best of our knowledge, the present study is the first to demonstrate that EBLN3P act as a novel oncogene in osteosarcoma. Furthermore, EBLN3P act as a ceRNA to regulate Rab10 expression via competitively sponging to miR-224-5p, thereby regulating the progression of osteosarcoma. These findings may provide useful information to identify new biomarkers for early diagnosis and therapeutic applications in osteosarcoma.

## Supplementary Information

Supplementary Information

## Data Availability

The datasets used and/or analyzed during the present study are available from the corresponding author on reasonable request.

## Abbreviations

ceRNA: Competes endogenous RNAs
Rab10: Endogenous bornavirus like nucleoprotein, EBLN3P ras related protein 10
HOTAIR: Hox transcript antisense intergenic RNA
lncRNA: Long non-coding RNAs
miRNAs: MicroRNAs
qRT‐PCR: Quantitative real‐time polymerase chain reaction
shRNA: Short hairpin RNA
