# Supplementary material for: LncRNA EBLN3P promotes the progression of osteosarcoma through modifying the miR-224-5p/Rab10 signaling axis
Source: Sci Rep. 2021 Jan 21;11:1992. doi: 10.1038/s41598-021-81641-6 (PMC7820338; doi:10.1038/s41598-021-81641-6)

LncRNA EBLN3P promotes the progression of osteosarcoma through modifying the miR-224-5p/Rab10 signaling axis

Shuhong Dai[1 &]: shuhong0404@163.com

Ning Li[2 &]: 18560294521@163.com

Ming Zhou[3]: zmzxyy@126.com

Yue Yuan[4]: yueyuan@jlu.edu.cn

Ding Yue[5]: 531471207@qq.com

Tao Li[3,6*]: taolichina@126.com

Xiaowei Zhang[3,6*]: zxw90@foxmail.com

[*] Corresponding author

[1] Department of Cardiac intensive care unit, Central Hospital of Zibo, Affiliated with Shandong University, Zibo, Shandong Province, China.

[2] Department of Combination of Chinese Traditional and Western Medicine, Central Hospital of Zibo, Affiliated with Shandong University, Zibo, Shandong Province, China

[3] Department of Orthopedic Surgery, Central Hospital of Zibo, Affiliated with Shandong University, Zibo, Shandong Province, China

[4] Experimental Center of Medical Biology, School of Basic Medical Sciences, Jilin University, Changchun, China

[5] Department of Pathogen biology, The Key Laboratory of Zoonosis, Chinese Ministry of Education, College of Basic Medicine, Jilin University, Changchun, Jilin, 130021, China

[6] Center for Translational Medicine, Central Hospital of Zibo, Affiliated with Shandong University, Zibo, Shandong Province, China

[&] These authors contributed equally to this work

The expression of β-actin in OS cell lines hFOB.1.19 Saos2 MG63 143B U2OS.

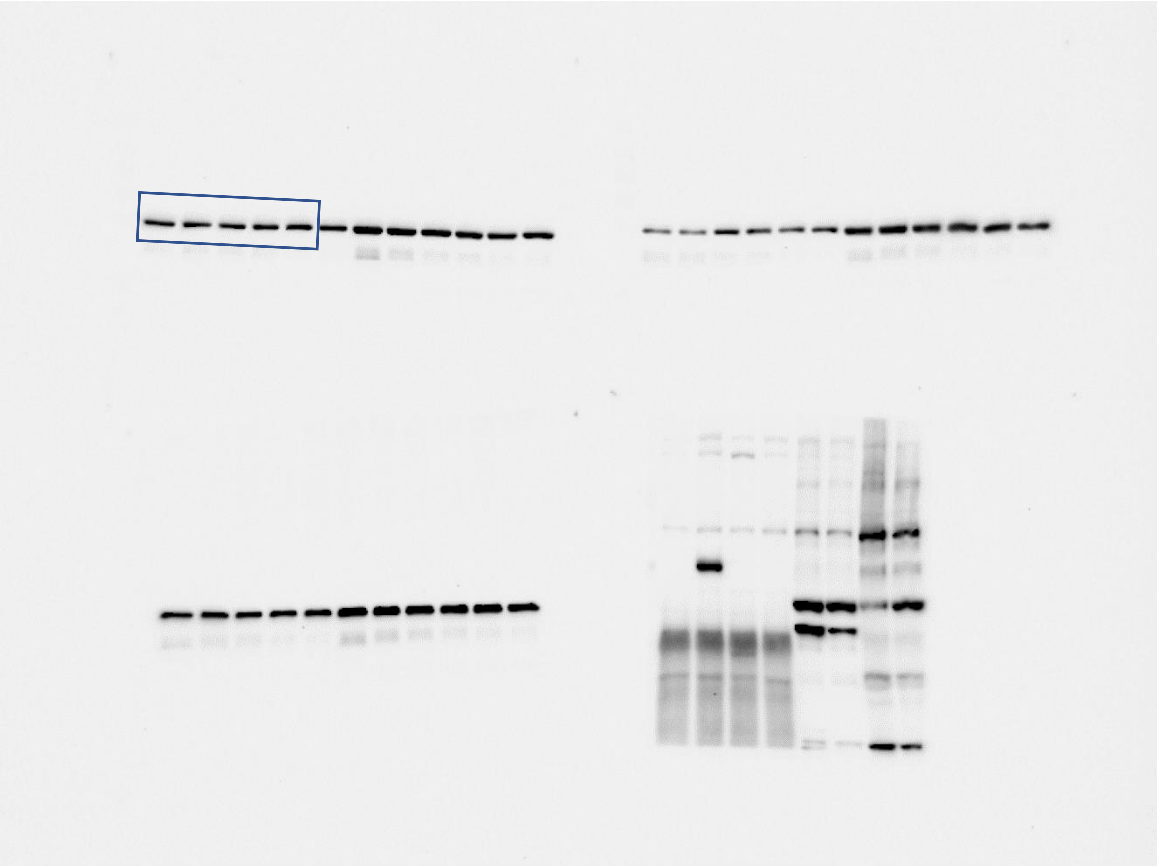

The expression of Rab10 in OS cell line hFOB.1.19 Saos2 MG63 143B U2OS.

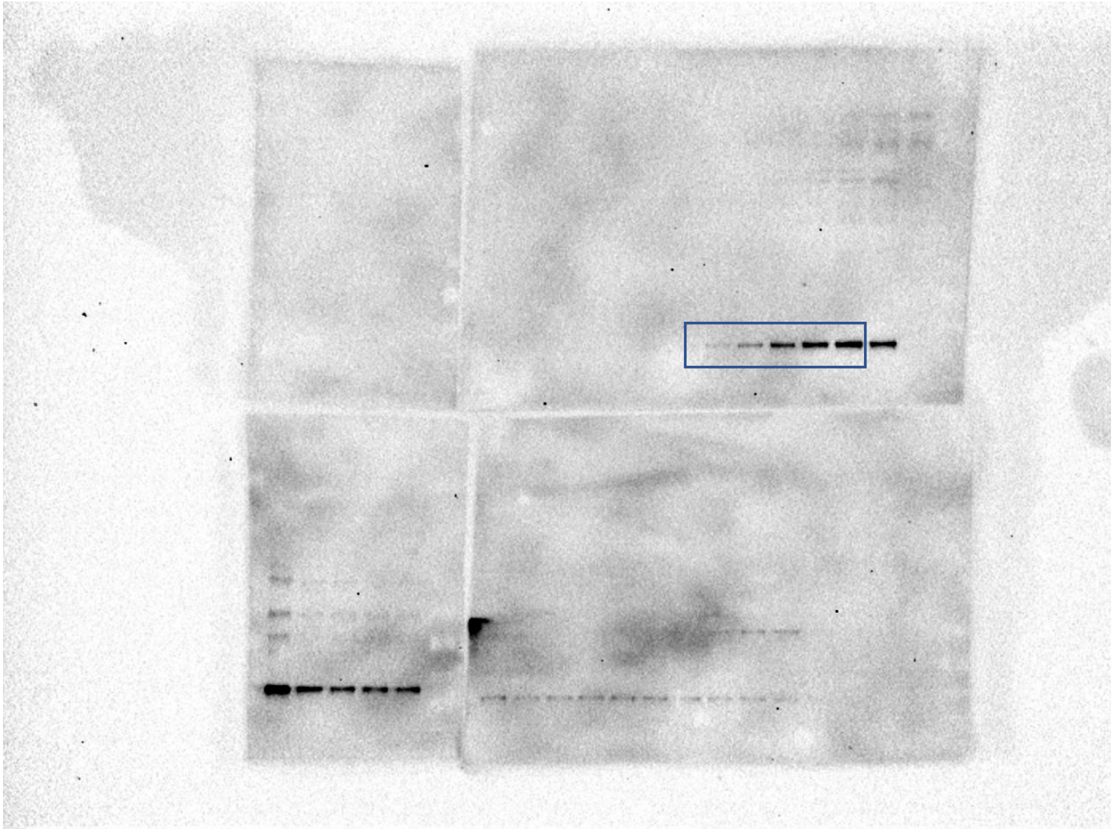

The expression of β-actin in OS tissues and negative control.

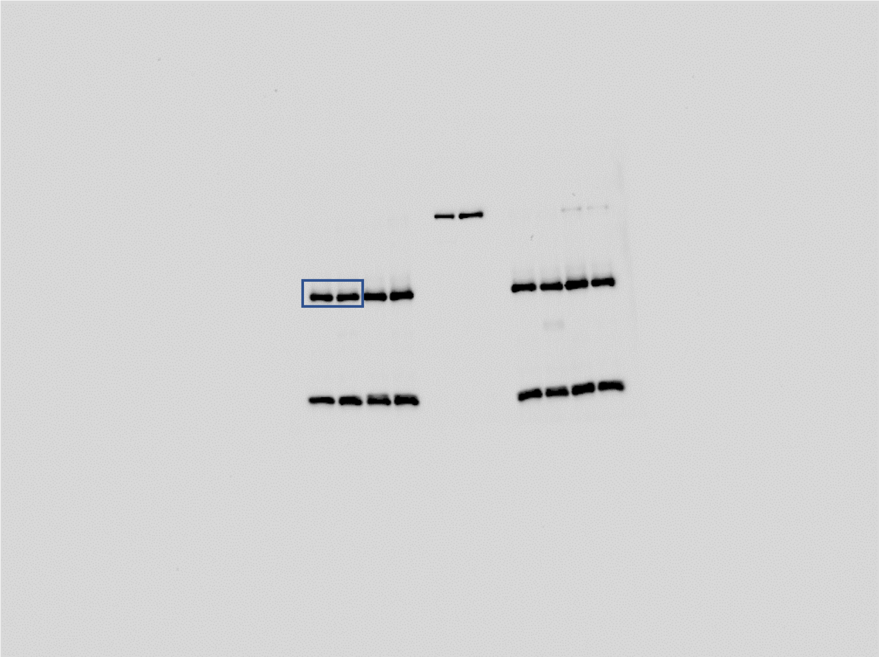

The expression of Rab10 in OS tissues and negative control.

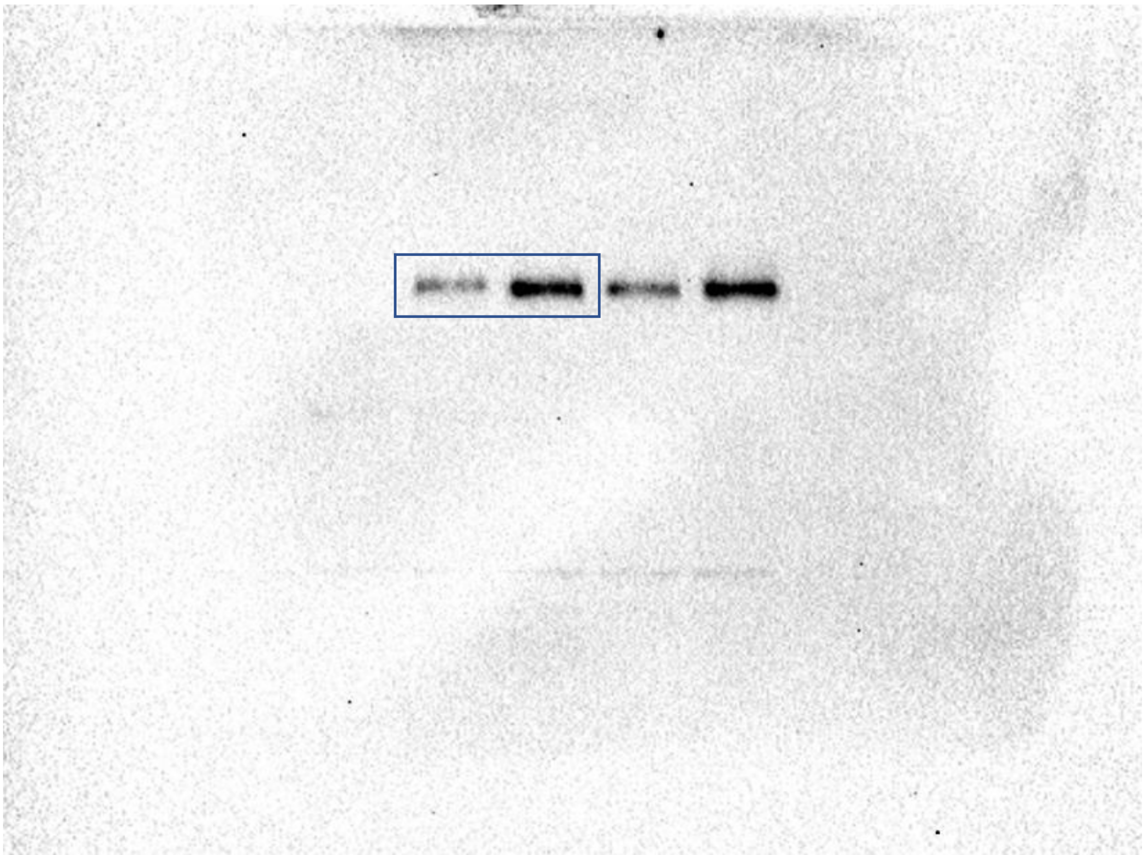

The expression of β-actin in mimics NC, miR-224-5p mimics, inhibitor NC, and miR-224-5p inhibitor group.

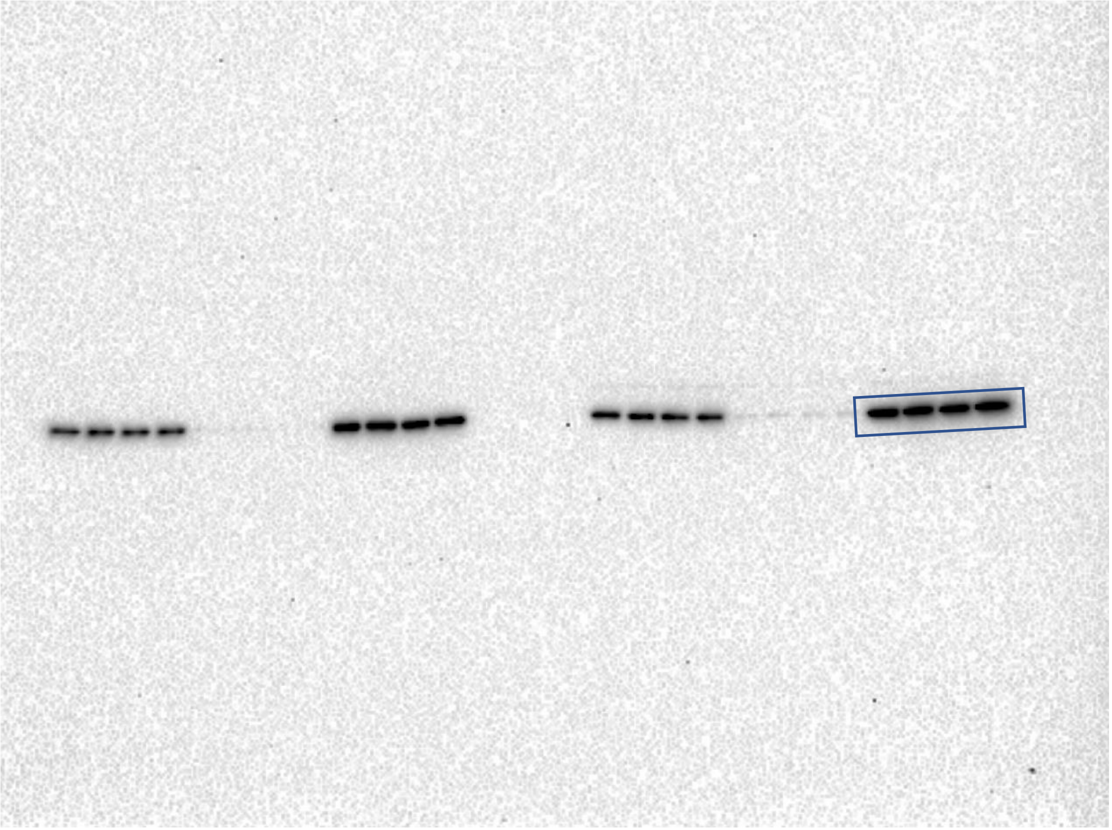

The expression of Rab10 in mimics NC, miR-224-5p mimics, inhibitor NC, and miR-224-5p inhibitor group.

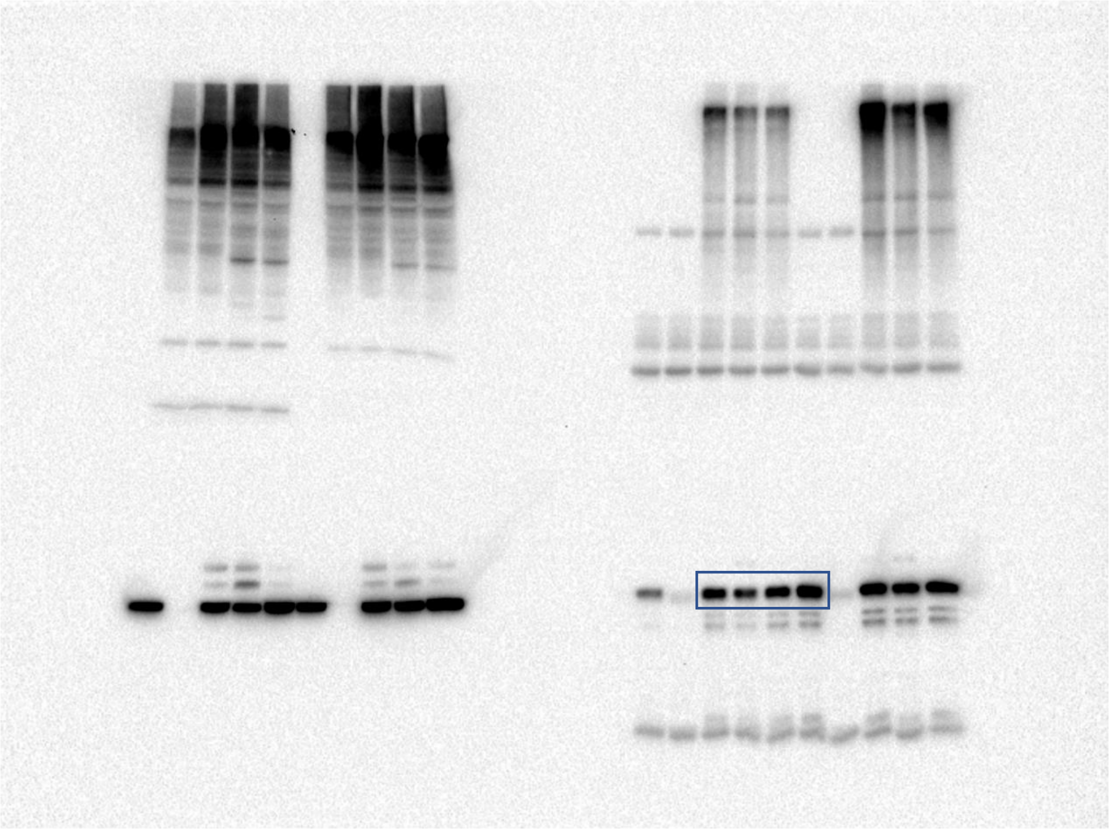

The expression of β -actin in si-EBLN3P+inhibitor NC, si-EBLN3P+miR inhibitor, si-EBLN3P+NC, si-EBLN3P+Rab10 group.

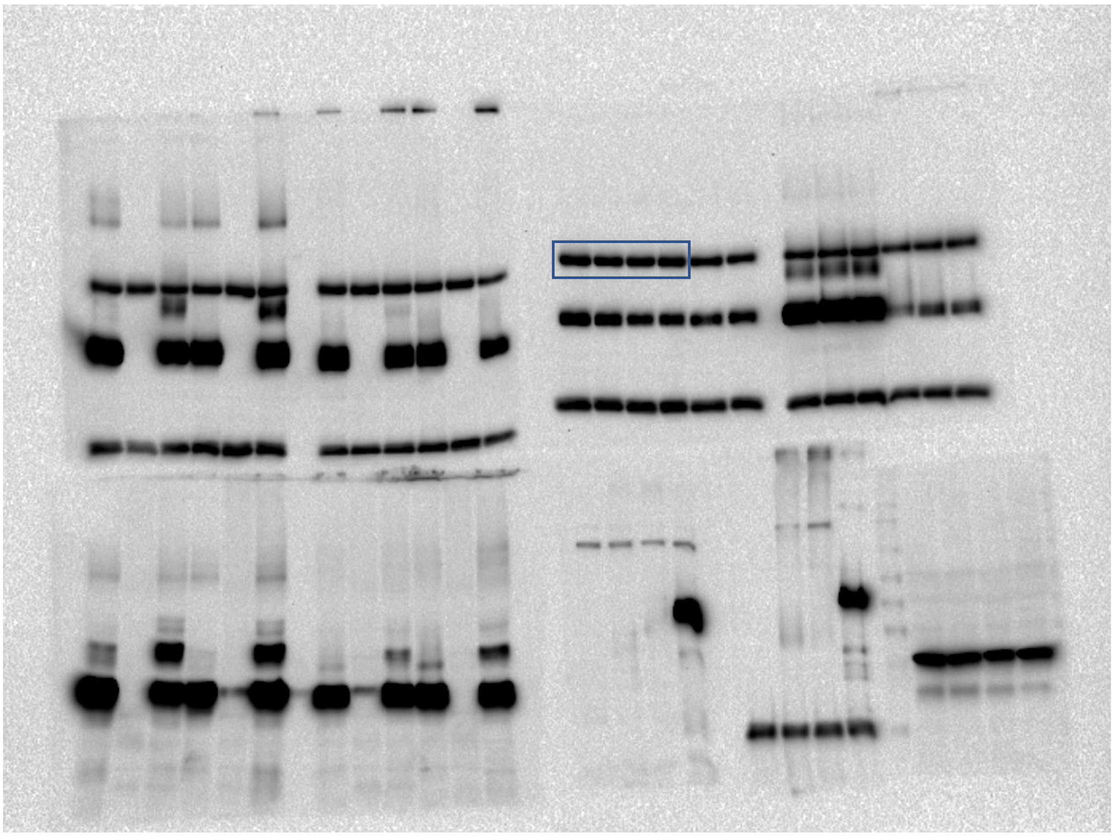

The expression of Rab10 in si-EBLN3P+inhibitor NC, si-EBLN3P+miR inhibitor, si-

EBLN3P+NC, si-EBLN3P+Rab10 group.

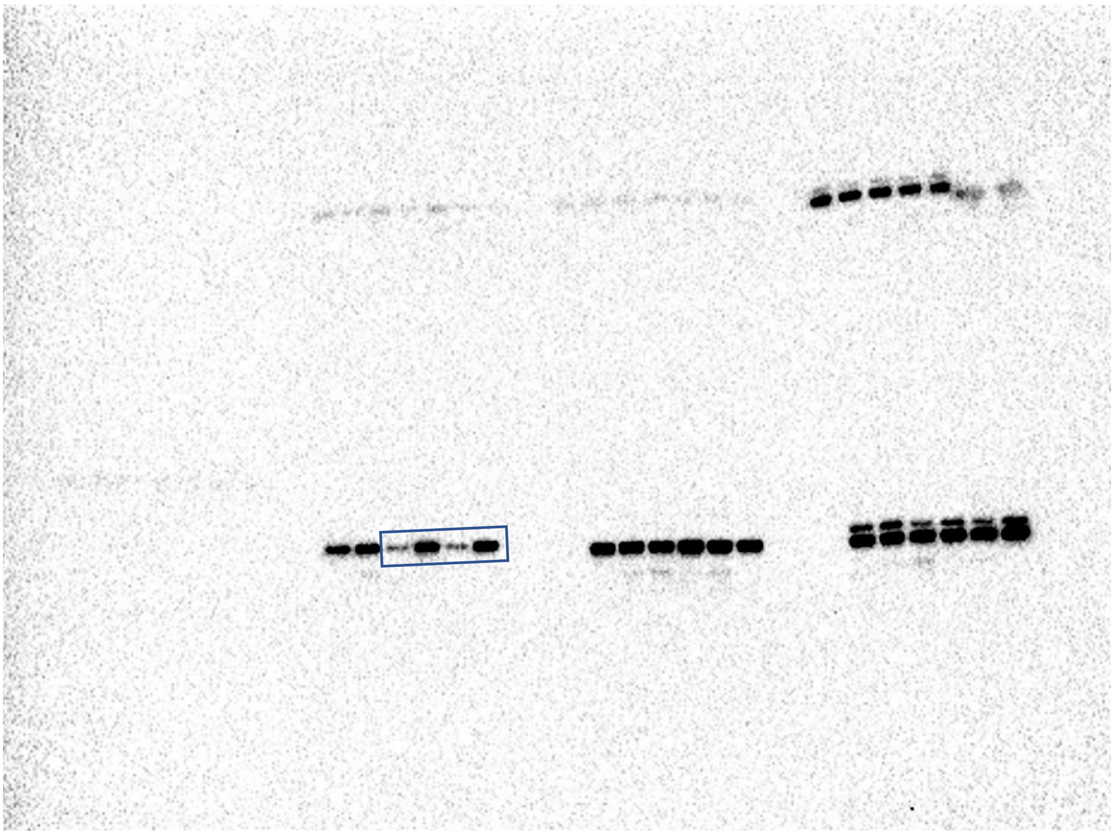

Supplement: Supplementary file 1 — Supplementary Information [file 41598_2021_81641_MOESM1_ESM.pdf]
